# Personal

**Published:** 1904-06

**Authors:** 


### PERSONAL.

Dr. Charles A. L. Reed, of Cincinnati, delivered the address at the annual commencement of the Army Medical School at Washington, April 5, 1904, choosing for his subject, The medical profession in the public and private life of America. It is almost needless to add that this unique subject was dealt with in a liberal and masterful fashion. The address is published in full in the *Journal of the American Medical Association*, May 14, 1904.

---

Dr. Edward N. Brush, superintendent of the Sheppard and Enoch Pratt Hospital, near Baltimore, visited Buffalo during the university commencement in May. He is president of the class of '74, which held a reunion on that occasion.

---

Dr. Chauncey Pelton Smith, who has been confined to his home for several weeks as the result of an accident in which his leg was fractured, has recovered and resumed his professional practice. He has removed from No. 481 Franklin street, to The Raleigh, No. 354 Franklin street. Hours: 9 to 10 a. m., 2 to 3 p. m. and 8 p. m. Telephone: Tupper 412.

Dr. William H. Thornton, of Buffalo, removed May 1, 1904, from 572 Niagara street to 231 Norwood avenue, corner of West Utica street. Hours: 8 to 9 a. m., 2 to 3 and 7 p. m. Sundays by appointment. Both telephones.

---

Dr. William Irving Thornton removed May 1, 1904, from 152 Jersey street to 572 Niagara street, the former office of Dr. William H. Thornton. Hours: 8 to 9 a. m., 2 to 3 and 7 to 8 p. m. Sundays, 9 a. m. and 2.30 p. m. Both telephones.

---

Dr. A. B. Knisley, of Buffalo, announces the removal of his office and residence to No. 56 Woodlawn avenue. Hours: 12.30 to 1.30 and 7 to 8 p. m. Telephones: Bell, Bryant 574; Frontier, 1602. Branch office, 360 Swan street. Hours: 2 to 4 p. m. Telephone: Bell, Howard 228.

---

Dr. John J. Twohey, of Buffalo, has removed his office and residence from No. 301 Masten street, to 2200 Main street. Telephone: Park 121.

---

Dr. David Wheeler, of Buffalo, has been appointed an attending physician at the Erie County Hospital in the department of genitourinary surgery.

---

Dr. J. MacDonald, Jr., of New York, secretary of the Medical Editors' Association, has received the degree of doctor in medicine from the Baltimore University School of Medicine.

---

Dr. W. E. Merriman, of Albany, has been appointed assistant surgeon of the Soldiers' and Sailors' Home at Bath, vice Dr. Cameron Haskell, promoted.

---

Dr. William P. Spratling, superintendent of the Craig Colony for Epileptics at Sonyea, has been appointed superintendent of Bellevue Hospital at New York.

---

Dr. Cameron Haskell has been promoted from assistant surgeon to chief surgeon at the Soldiers' and Sailors' Home, at Bath, vice Dr. W. L. Babcock, resigned.

---

Dr. William Mabon, of New York, superintendent of Bellevue Hospital, has been appointed by Governor Odell, president of the State Commission in Lunacy, vice Dr. Frederick Peterson, resigned.

---

Dr. Robert P. Bush, of Horseheads, N. Y., former speaker of the Assembly, attended the annual commencement of Buffalo University in May. He was elected a member of the board of trustees of the Alumni Association.

---

Dr. Lee A. Whitney, of Buffalo, has been appointed from the state civil service eligible list to be resident physician at the State Hospital for crippled and deformed children at Tarrytown, at a salary of $900 a year.

---

Dr. Roswell Park, of Buffalo, was elected president of the Liberal Club, at its meeting held May 6, 1904. The Liberal and Independent clubs were consolidated and Dr. Park becomes president of the united organisation, this action having been taken while he was on the way home from Europe.

---

Dr. Edward E. Koehler, of Buffalo, announces the removal of his office and residence from 696 Broadway, to 715 Broadway, corner Krettner street.

---

Dr. John S. Halbert, of Buffalo, who has lived at 459 Franklin street for eight years, has leased his house and removed to the old family homestead, 2485 Main street.

---

Dr. F. W. McGuire, of Buffalo, announces the removal of his office and residence to 1175 Main street, corner of Dodge. Hours: 8 to 9 a. m., 1 to 3 and 7 to 8 p. m. Telephone: Bryant 144.

---

Dr. Harry R. Trick, of Buffalo, has leased the offices of the late Dr. C. C. Wyckoff, 482 Delaware avenue.

---

Dr. Warren L. Babcock, chief surgeon at the State Soldiers' Home Hospital, Bath, N. Y., resigned May 12, 1904, to accept the superintendency of Grace Hospital, Detroit, Mich.

---

Dr. E. R. McGuire announces the removal of his office and residence to 1175 Main street, corner of Dodge. Hours: 8 to 9 a. m., 1.30 to 2.30 and 7 to 8 p. m. Telephone: Bryant 144.

